# Increasing physical work capacity losses due to heat stress increase

**DOI:** 10.1007/s00484-025-03008-0

**Published:** 2025-08-15

**Authors:** Seok-Geun Oh, Seok-Woo Son, Dong-Chan Hong

**Affiliations:** 1 School of Earth and Environmental Sciences, Seoul National University, Seoul, South Korea; 2 Climate Technology Center, Seoul National University, Seoul, South Korea

**Keywords:** Heat stress days, Physical work capacity loss, Global warming

## Abstract

As global warming continues, hotter and more humid weather conditions become increasingly frequent, heightening population exposure to heat-stress environments. This could lead to a loss of physical work capacity (PWC loss) and result in significant economic risks. Here, we investigate the PWC loss risks and their recent changes under different heat stress environments for the period of 1985–2023. Changes in PWC loss risks on comfort days, defined by heat stress values of 20–29 °C, show a latitudinal contrast, with an overall decrease in low latitudes and an increase in high latitudes. However, they have increased everywhere on both discomfort days (30–45 °C) and dangerous days (above 45 °C), with a greater increase in the latter condition. This suggests that global warming is exacerbating the risks of PWC loss in more intense heat stress environments. Geographically, Western Europe, the Mediterranean, Siberia, the Caribbean, and northeastern South America have experienced more significant increases in PWC loss risks, exceeding 1.5 times relative to the climatological mean over the past four decades. Such changes are due more to changes in the frequency of heat stress days than to changes in the severity of PWC loss. This finding suggests that the spatio-temporal variations in meteorological aspects should be taken into account when designing policies to address PWC loss risks due to heat stress.

**Supplementary Information:**

The online version contains supplementary material available at 10.1007/s00484-025-03008-0.

## Introduction

As global warming continues, hot and humid weather conditions become more frequent and intense, occasionally exceeding human thermoregulatory limits and threatening human life and activities (Dunne et al. 2013; Zhu et al. 2021). Exposure to such heat-stress environments significantly reduces work capacity (i.e., decrease in productive working time) and results in substantial economic losses (Rezai et al. 2018; Zhang et al. 2018). In 2004, the loss of work capacity due to heat stress was 5.52 billion euros per year in Australia (Zander et al. 2015), and between 686.64 million euros and 3.02 billion euros in Germany (Hübler et al. 2008). Continued global warming is expected to lead to more frequent and intense heat-stress events (Dunne et al. 2013; Zhu et al. 2021; Nelson et al. 2023). This has the potential to further exacerbate work capacity losses and ultimately increase social and economic risks.

Physical work capacity (PWC) refers to the maximum level of physical effort that an individual can sustain over an extended period of time without experiencing undue fatigue or health risks (Foster et al. 2021). Numerous models have been proposed and applied to estimate PWC losses and their economic impact (e.g., Dunne et al. 2013; Zivin and Neidell 2014; Kjellstrom et al. 2018). These studies have mainly focused on the regional scale. Global applications are rare due to several factors such as regionally biased data, overestimation of heat-acclimated populations, and a limited set of heat stress indicators. Recently, Foster et al. (2021) proposed an advanced PWC model that can be applied globally across industries. Since then, a number of studies have used their method to quantify PWC losses for specific regions or industries (Dawkins et al. 2023; Nelson et al. 2023). However, the potential economic risks associated with PWC losses due to heat stress in a warming climate remain largely unexplored. Such study is critical for designing efficient development strategies and ensuring a sustainable future, as heat-stress-related PWC losses directly impact national productivity, individual incomes, and sustainable development goals (Vanos et al. 2020; Matsumoto et al. 2021; Nelson et al. 2023).

Here, we investigate the spatio-temporal changes in the potential economic risks of heat-stress-related PWC losses (hereafter, PWC loss risks) under different heat stress environments for the period of 1985–2023. Using hourly 2-m air temperature and relative humidity data, we calculate the frequency of heat stress days at different levels, the severity of PWC losses due to heat stress, and the PWC loss risks that integrate heat stress days and PWC loss severity. We then characterize the spatio-temporal changes in PWC loss risks by comparing the last 10 years (2014–2023) with the first 10 years of the analysis period (1985–1994). As shown below, global warming is exacerbating PWC loss risks in more intense heat-stress environments.

## Data and methods

### Climate data

Hourly 2-m air temperature and relative humidity data are obtained from the fifth generation of the European Centre for Medium-Range Weather Forecast global reanalysis (ERA5, Hersbach et al. 2020). They are used to calculate the heat stress conditions for the period of 1985–2023. The ERA5 data has a higher spatial (30 km) and temporal (hourly) resolution than other reanalysis data (e.g., JRA-55, Kobayashi et al. 2015), making it more suitable for characterizing regional climate extremes and for evaluating climate models (e.g., Li et al. 2021; Oh et al. 2024).

### Heat stress metric: humidex

Heat stress is quantified using the Humidex. Humidex provides a simple yet effective way to combine air temperature and relative humidity into a single value that reflects human-perceived heat stress (Masterton and Richardson 1979; Kennedy-Asser et al. 2021; Dawkins et al. 2023). This index is widely used in public health and occupational studies and fits well with our focus on PWC losses (e.g., Li et al. 2023; Ferrari et al. 2025). More sophisticated indices have recently been proposed. An example is the wet-bulb globe temperature (WBGT; Davis et al. 2024), which considers solar radiation and wind speed in addition to air temperature and relative humidity. However, when applied to climate models especially for future projections, WBGT or a similar index has a large uncertainty because solar radiation and wind speed are poorly constrained in climate models compared to air temperature and relative humidity (Xu et al. 2021; Shen et al. 2022; He et al. 2023; Zha et al. 2023; Francis and Fonseca 2024).

Humidex is computed with 2-m air temperature (Ta, °C) and relative humidity (RH, %) as follows;

1 $$\:\text{H}\text{u}\text{m}\text{i}\text{d}\text{e}\text{x}\left({}^\circ\mathrm C\right)=\text{T}\text{a}+\frac59\left(6.112\times\:10^{\left(\frac{7.5\text{T}\text{a}}{237.7+\text{T}\text{a}}\right)}\times\:\frac{\text{R}\text{H}}{100}-10\right).$$

Humidex provides a number that describes how humans feel thermal discomfort (e.g., Kennedy-Asser et al. 2021; Dawkins et al. 2023). As summarized in Table 1, Humidex values between 20 and 29 °C represent little to no discomfort. In contrast, values between 30 and 39 °C represent some discomfort, while those between 40 and 45 °C represent severe discomfort. Humidex values exceeding 45 °C are considered dangerous and pose a significant risk of heat stroke. To account for the maximum heat stress effects on humans, a daily maximum temperature extracted from the hourly 2-m air temperature at each grid is used in Eq. (1), along with the corresponding relative humidity.

**Table 1:** Ranges of the humidex index corresponding to rising thermal discomfort conditions

Humidex range	Thermal discomfort level	Event definition in this study	
20 °C ≤ Humidex ≤ 29 °C	Comfort		Comfort days
30 °C ≤ Humidex ≤ 39 °C	Some discomfort		Discomfort days
39 °C ≤ Humidex ≤ 45 °C	Great discomfort, avoid exertion		
Above 45 °C	Dangerous		Dangerous days

### PWC loss severity and risk definition

Vulnerability refers to the susceptibility of exposed people or assets to a hazard, often characterized by parameters derived from exposure data outlined in the risk assessment (Dawkins et al. 2023). In this study, the Humidex-based vulnerability function, proposed by Foster et al. (2021), is used to define the PWC. This function is based on 338 work sessions in climatic chambers (low air movement, no solar radiation) ranging from mild to extreme heat stress. The PWC is quantified as below:

2 $$\:\text{P}\text{W}\text{C}\:\left(\text{u}\text{n}\text{i}\text{t}:\:{\%}\right)=\frac{100}{1+{\left(\frac{\text{p}1}{\text{H}\text{u}\text{m}\text{i}\text{d}\text{e}\text{x}}\right)}^{\text{p}2}},$$

where p1 is the Humidex value that induces 50% PWC, estimated to be 54.5 °C, and p2 is the slope of the vulnerability function (i.e., the increase in reduced PWC per °C increase in Humidex), estimated to be −4.1% per °C. This equation is inverted to relate the Humidex to the PWC loss. The result is then scaled to a range of 0 to 1 by dividing by 100.

3 $$\mathrm{PWC}\;\mathrm{loss}=\left(100-\mathrm{PWC}\right)/100$$

The PWC loss risk is estimated by combining heat stress events and severity of the PWC loss:

4 $$\begin{aligned}&\:\text{P}\text{W}\text{C}\:\text{l}\text{o}\text{s}\text{s}\:\text{r}\text{i}\text{s}\text{k}\:(\text{u}\text{n}\text{i}\text{t}:\:\text{P}\text{W}\text{C}\:\text{l}\text{o}\text{s}\text{s}\:\text{d}\text{a}\text{y}\text{s})=\\&\text{H}\text{e}\text{a}\text{t}\:\text{s}\text{t}\text{r}\text{e}\text{s}\text{s}\:\text{e}\text{v}\text{e}\text{n}\text{t}\:\text{p}\text{r}\text{o}\text{b}\text{a}\text{b}\text{i}\text{l}\text{i}\text{t}\text{y}\times\:\text{P}\text{W}\text{C}\:\text{l}\text{o}\text{s}\text{s}\:\text{s}\text{e}\text{v}\text{e}\text{r}\text{i}\text{t}\text{y}. \end{aligned}$$

The PWC loss severity is calculated by weighting the vulnerability with the exposure:

5 $$\begin{aligned}&\:\text{P}\text{W}\text{C}\:\text{l}\text{o}\text{s}\text{s}\:\text{s}\text{e}\text{v}\text{e}\text{r}\text{i}\text{t}\text{y}\:\left(\text{u}\text{n}\text{i}\text{t}:\:\text{P}\text{W}\text{C}\:\text{l}\text{o}\text{s}\text{s}\:\text{d}\text{a}\text{y}\text{s}\right)=\\&\text{E}\text{x}\text{p}\text{o}\text{s}\text{u}\text{r}\text{e}\times\:\text{V}\text{u}\text{l}\text{n}\text{e}\text{r}\text{a}\text{b}\text{i}\text{l}\text{i}\text{t}\text{y}=\text{P}\text{o}\text{p}\text{u}\text{l}\text{a}\text{t}\text{i}\text{o}\text{n}\times\:\text{P}\text{W}\text{C}\:\text{l}\text{o}\text{s}\text{s}. \end{aligned}$$

The exposure is quantified by the population in 2010 at a given grid (Jones and O’Neill 2016), whereas the vulnerability is measured by PWC loss (Eq. 3). Assuming a person works one day in optimal conditions, the severity physically represents the number of working days lost due to heat stress events. Its unit is PWC loss days.

The PWC loss risks are assessed under the three different heat stress environments. Based on the range of the Humidex value (Table 1), heat stress events are categorized into comfort, discomfort, and dangerous days. For each category, the PWC loss risks are quantified by combining the severity of the event with its probability of occurrence, expressed in terms of PWC loss days.

Through this analysis, the present study aims to understand the spatio-temporal changes in the potential economic risks of PWC loss due to global warming at different heat stress levels, and to provide the reference for future scenario analysis based on climate models.

## Results

**Fig. 1:**
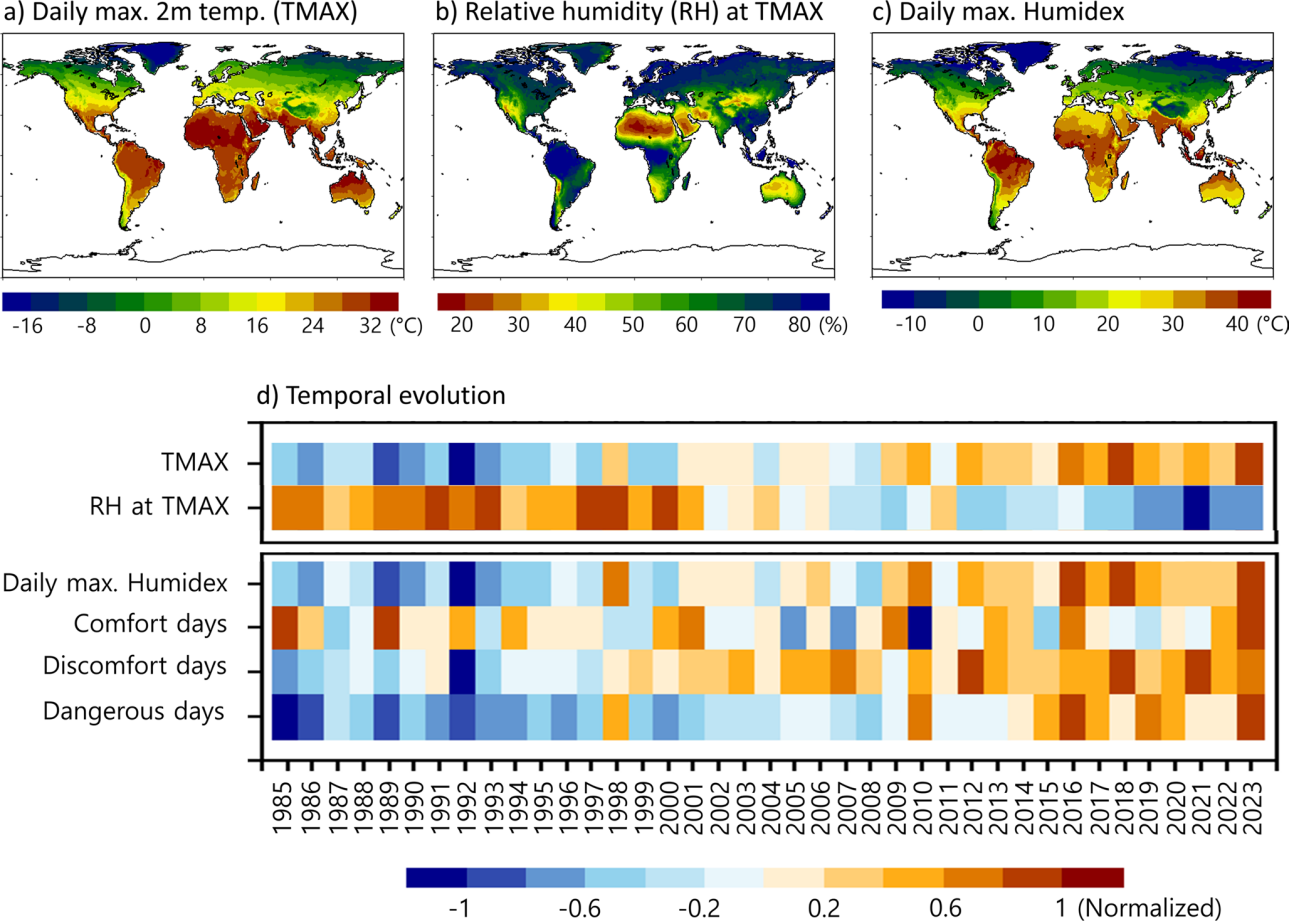
Spatial distribution of annual mean daily maximum 2-m air temperature (TMAX), relative humidity (RH) corresponding to TMAX, and daily maximum Humidex for the period of 1985–2023 and their globally averaged time series. The time series of comfort days, discomfort days, and dangerous days, calculated from daily maximum Humidex-based thresholds (see Table 1) are also presented. Only land grids are used for the time series analysis, and the statistical values of each variable are normalized through the min-max scaling

The global distribution of daily maximum 2-m air temperature (hereafter temperature) is influenced by both latitude and topography, with temperature typically decreasing at higher latitudes and altitudes (Fig. 1a). The spatial distribution of relative humidity generally exhibits an inverse relationship with temperature (Fig. 1b). Humidex appears to follow the distribution of temperature more closely than that of relative humidity (Fig. 1c). This indicates that although Humidex is determined by both temperature and relative humidity (Eq. 1), it is more strongly influenced by temperature with a secondary contribution from relative humidity (Masterton and Richardson 1979; Kennedy-Asser et al. 2021; Dawkins et al. 2023).

The time series of land-averaged temperature and relative humidity are shown in Fig. 1d. The time series of Humidex and the three levels of heat stress days (comfort, discomfort, and dangerous days), derived from temperature and relative humidity, are also presented. All of them are normalized by maximum-minimum values. It is evident that land temperature has steadily increased, while relative humidity has steadily decreased (first and second rows in Fig. 1d). The decrease in relative humidity over the land is mainly due to the decrease in soil moisture with anthropogenic warming (Zhou et al. 2023). Humidex has also increased steadily as in land temperature. Since 2010, Humidex has been consistently above its climatology, and its inter-annual variability is remarkably similar to that of the land temperature, with a temporal correlation of 0.97 (third row in Fig. 1d).

There is a consistent upward trend in the number of discomfort days and dangerous days (fifth and sixth rows in Fig. 1d). In particular, the number of dangerous days has risen sharply since the mid-2010s. However, the number of comfort days does not show a distinct trend (fourth row in Fig. 1d). This is due to the offsetting effects of the spatially contrasting changes in comfort days, as discussed later.

### Climatological distribution of PWC loss risks

**Fig. 2:**
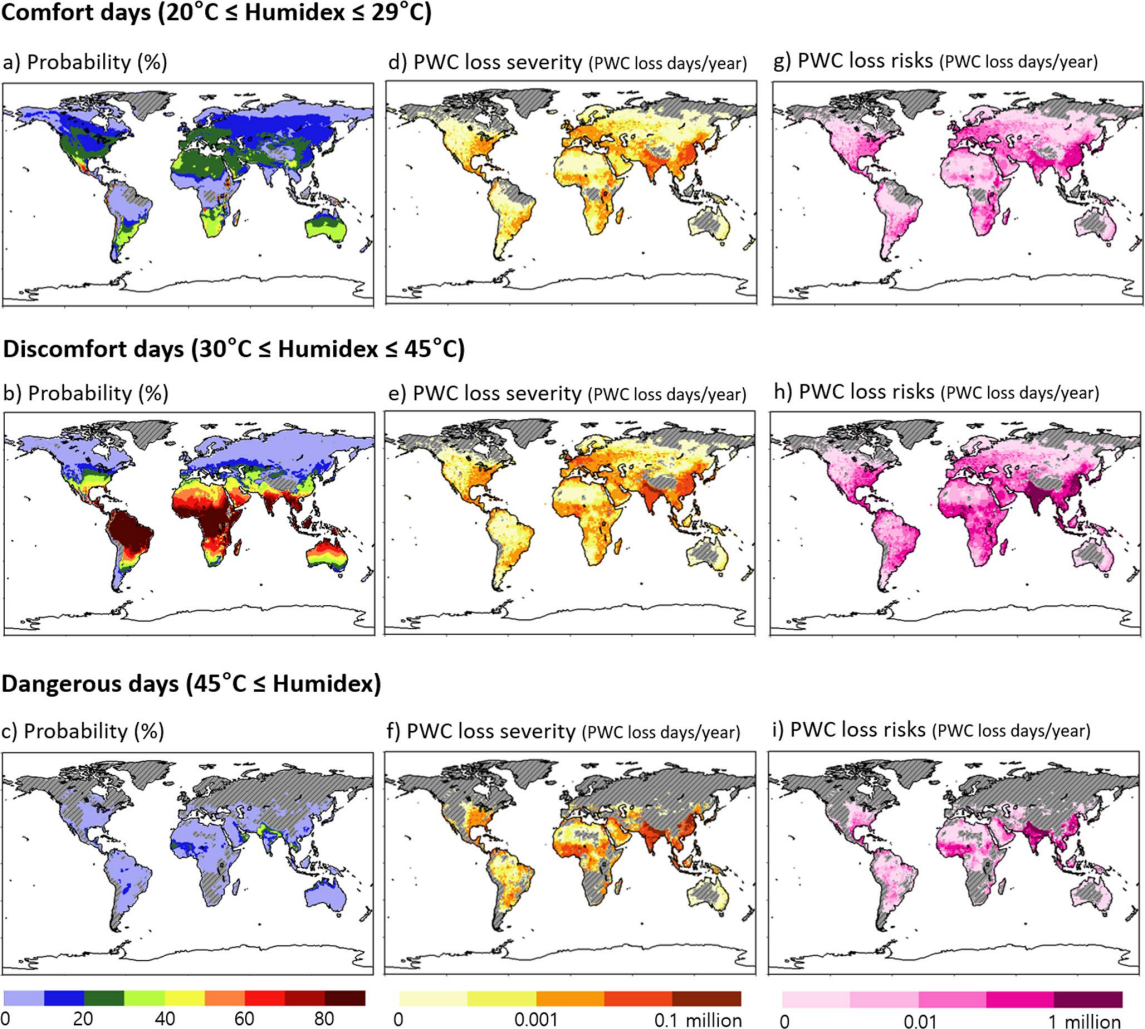
Spatial distribution of (**a**–**c**) probability of heat stress event occurrence, (**d**–**f**) physical work capacity (PWC) loss severity and (**g**–**i**) PWC loss risk, for comfort days, discomfort days, and dangerous days for the period of 1985–2023. Grey-shading denotes the region where each event does not occur

Figure 2 shows the climatological distribution of the probability of heat stress event occurrence, PWC loss severity, and PWC loss risks for the comfort, discomfort, and dangerous days for the period of 1985–2023. Comfort days are widely distributed over much of the world, although they are relatively more frequent in mid-latitude regions with a probability of 20–40% (Fig. 2a). The occurrence of discomfort days is concentrated in low-latitude regions, with people residing in South America, Central America, and South Asia experiencing thermal discomfort for approximately 80% of the year (Fig. 2b). Dangerous days are relatively rare, compared to the other two cases, with a probability of less than 10% from low to mid latitudes (Fig. 2c). They are almost non-existent in the Northern Hemisphere high latitudes, although some parts of South Asia, such as Pakistan, Nepal, and Thailand, experience extreme heat stress for 30–50% of the year.

The PWC loss severity is generally more pronounced in densely populated areas (Eq. 5), particularly in Asia. It is observed to be higher in more intense heat-stress environments (Figs. 2d–f). In India, for example, PWC losses on comfort days range from 1,000 to 10,000 days. However, those on discomfort and dangerous days exceed 10,000 days everywhere, with a greater increase in areas where PWC losses exceed 100,000 days, particularly on dangerous days. This suggests that the more severe the heat stress, the greater the decline in work productivity, ultimately leading to greater potential economic losses.

The PWC loss risks due to heat stress manifest differently from its severity because of differences in relative occurrence probability of heat stress days (Figs. 2g–i). In India where discomfort days are relatively more frequent, PWC loss risks on discomfort days are about ten times higher than those on dangerous days (see India in Fig. 2h and i). Similarly, in Europe where comfort days are relatively more frequent than discomfort days, PWC loss risks on both comfort and discomfort days are quantitatively similar, ranging from about 1000 days to 1 million days (see Europe in Fig. 2g and h). This implies the need to consider not only social factors, such as population distribution in each country, but also meteorological factors, such as variations in heat stress days according to the geographical location, when developing policies to minimize PWC loss risks.

### Long-term changes of PWC loss risks

**Fig. 3:**
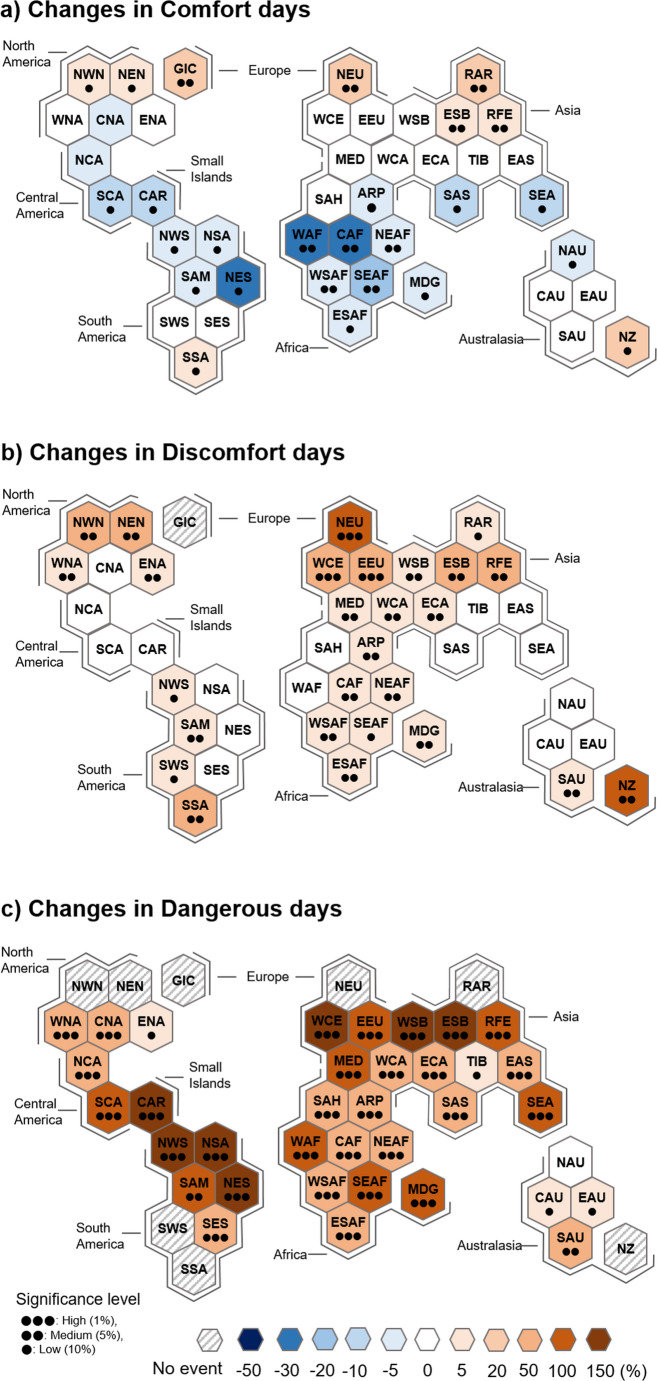
Regional changes of the probability of occurrence in the comfort, discomfort, and dangerous days for the period of 2014–2023 against the period of 1985–1994. This result is calculated as the difference in PWC loss risk between the latter and former 10-year periods, normalized by the climatological mean over the analysis period and expressed as a percentage. The three-letter codes in the hexagons show climate reference regions used in subcontinental climate analysis for the Intergovernmental Panel on Climate Change (IPCC) Special Report (Iturbide et al. 2020)

Spatio-temporal changes in comfort, discomfort, and dangerous days from the late 20th century (1985–1994) to the early 21 st century (2014–2023) are first examined in Fig. 3. To support regional policy development, they are quantified for 44 regions over the globe. Comfort days show a contrasting latitudinal change. They largely decrease in the South American, African, and South Asian regions (see NES, WAF, CAF, SAS, and SEA in Fig. 3a), but increase in the North American and Eurasian regions (see NWN, NEN, NEU, RAR, and REF etc.). These contrasting regional changes offset each other and explain the lack of a clear trend in comfort days over the past four decades (fourth row in Fig. 1d). In contrast, the distinct increase in discomfort and dangerous days (fifth and sixth rows in Fig. 1d) is found in most regions (Figs. 3b and c), with the increase in dangerous days being particularly pronounced.

**Fig. 4:**
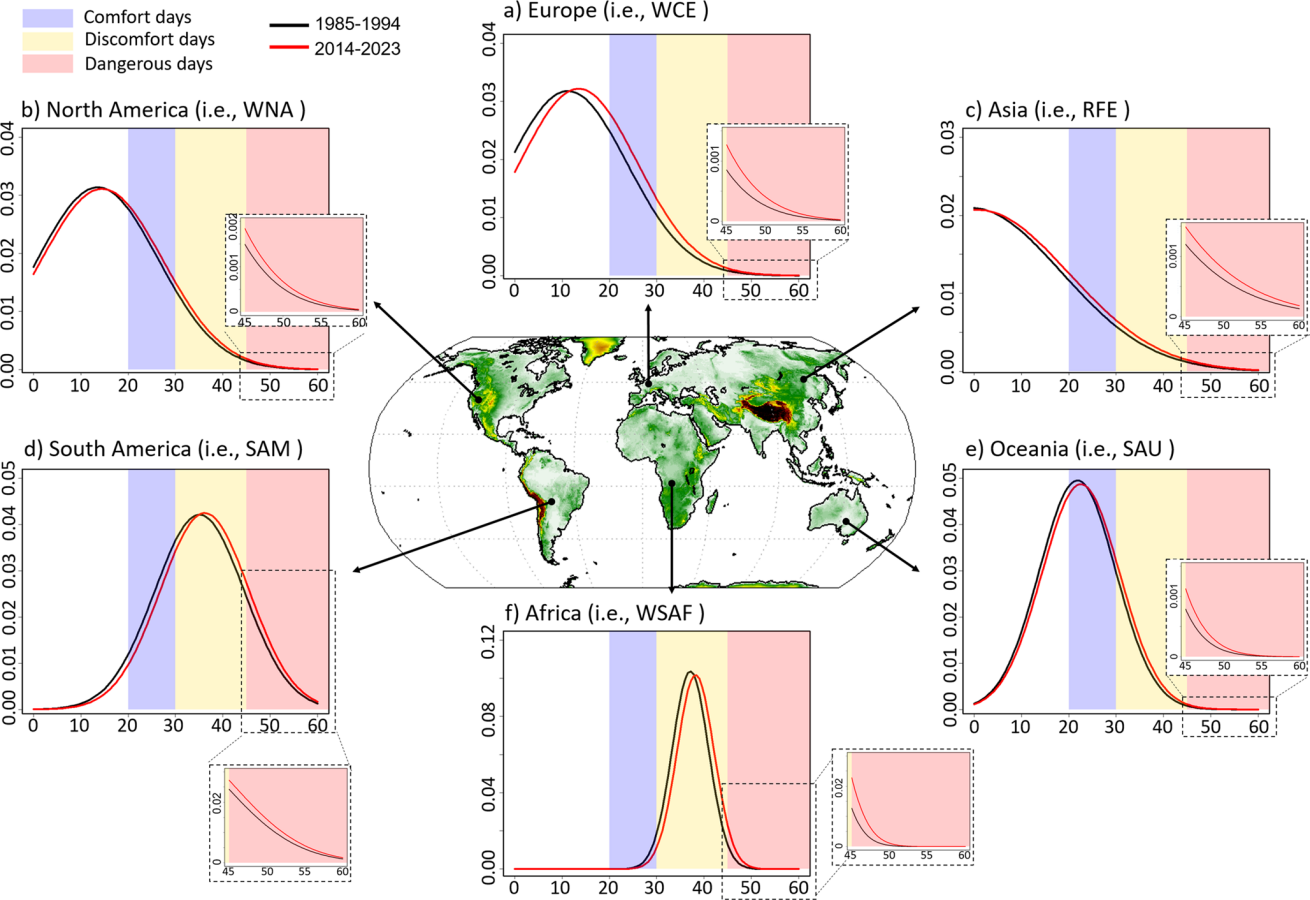
Probability density function of Humidex value in the selected sub-region of each continent for the period of 1985–1994 and 2014–2023

There is a clear warming shift in all regions when comparing the Humidex probability density distribution for the two analysis periods (Fig. 4). As Humidex increases in low-latitude regions such as South America, Africa, and South Asia due to global warming, heat stress days are more likely to occur in discomfort or dangerous conditions than in comfort conditions (Figs. 3 and 4). On the contrary, high-latitude regions such as North America and Eurasia are likely to experience an additional increase in warm weather conditions, i.e., comfort days (Figs. 3 and 4), due to global warming, which may provide benefits for outdoor activities (Choi et al. 2024). This is an example of the uneven impact of climate change on heat-related conditions.

**Fig. 5:**
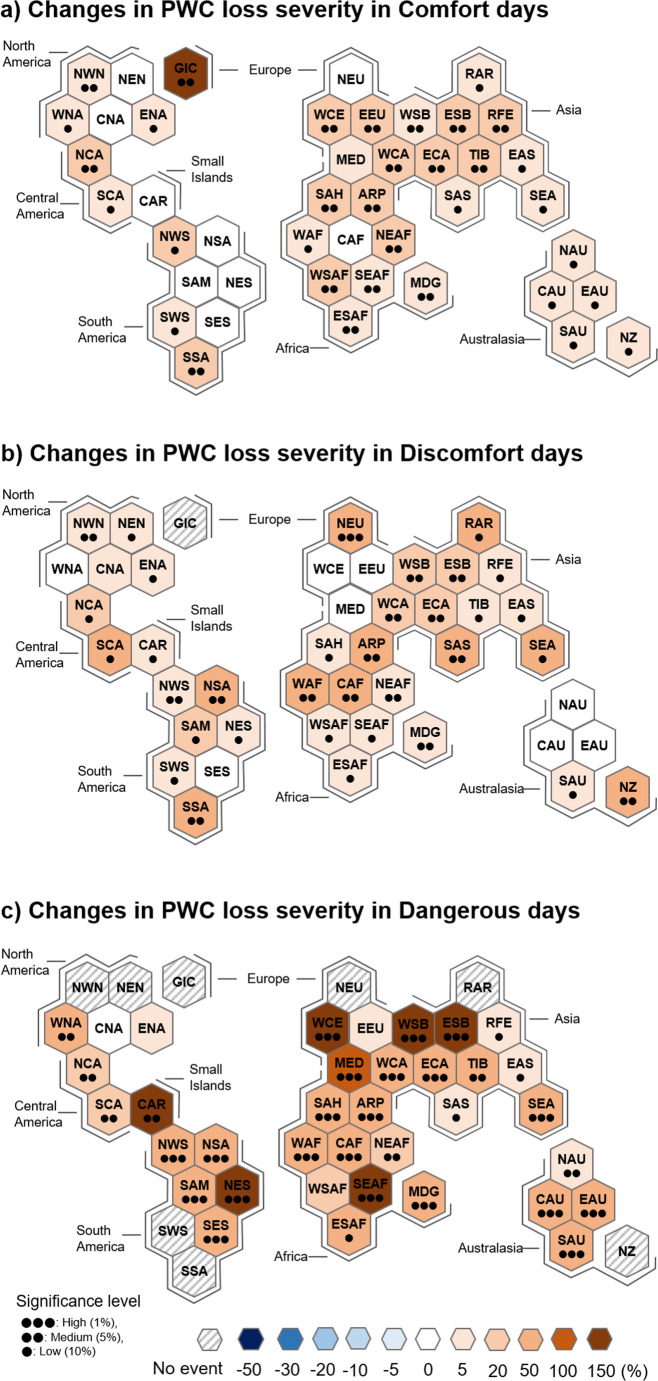
Same as Fig. 3 but for the PWC loss severity

Over the past four decades, the PWC loss severity increases for all heat stress days (Fig. 5), suggesting greater PWC losses in the 21 st century than in the last century due to global warming. Overall, the PWC loss severity has increased more significantly under more intense heat stress conditions. However, it is notable that the increase in the frequency of dangerous days is more pronounced than the increase in PWC loss severity on those days (compare Figs. 3c and 5c).

**Fig. 6:**
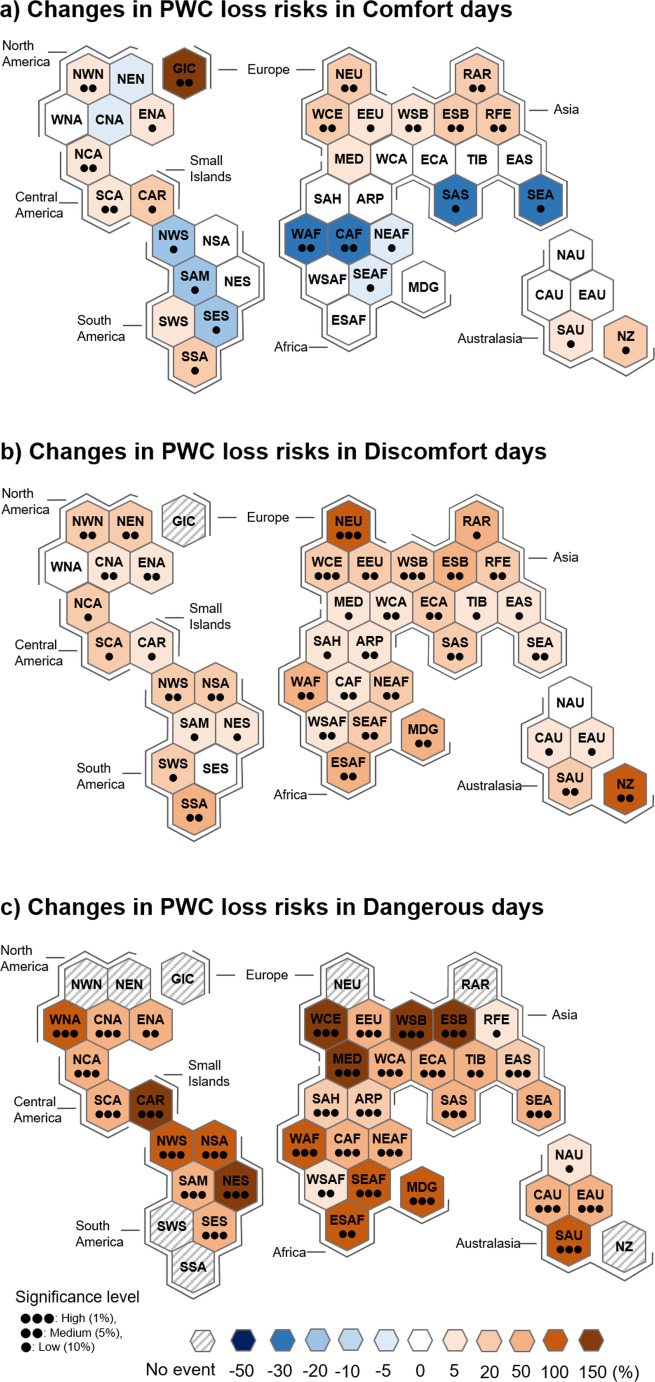
Same as Fig. 3 but for the PWC loss risks

Figure 6 shows the changes in PWC loss risks. It is evident that the changes in PWC loss risks are due more to changes in the frequency of heat stress days than to changes in the PWC loss severity (compare Fig. 6 with Figs. 3 and 5). For example, the PWC loss risks on comfort days show a contrasting latitudinal change (Fig. 6a), as in the changes in comfort days (Fig. 3a). The increase in PWC loss risks is greater in more intense heat-stress environments. Specifically, Western Europe, the Mediterranean, Siberia, the Caribbean, and northeastern South America have experienced a significant increase in the PWC loss risks on dangerous days, more than 150% (1.5 times) relative to the climatological mean (Fig. 6c). Similarly, Africa, northern South America, western North America, and southern Australia have seen risk increases of around 100%. This result indicates that the PWC loss risks could increase further in more intense heat-stress environments due to continued global warming, suggesting an urgent need for adaptation measures to mitigate the impact of heat stress on both human health and economic productivity.

## Summary and discussion

The present study investigates the PWC loss risks and their recent changes under the three different heat-stress environments, i.e., the Humidex-based comfort, discomfort, and dangerous days, for the period of 1985–2023. Changes in PWC loss risks on comfort days show a latitudinal contrast, with an overall decrease in low latitudes and an increase in high latitudes. It is also clear that the increase in PWC loss risks is greater on dangerous days than on discomfort days. Geographically, Western Europe, the Mediterranean, Siberia, the Caribbean, and northeastern South America have experienced more significant increase in PWC loss risks on dangerous days, exceeding 150% (1.5 times) over the past four decades. This result indicates that PWC loss risks could further increase in more intense heat-stress environments due to ongoing global warming.

The spatio-temporal changes in PWC loss risks are more significantly influenced by changes in heat stress days than those in PWC loss severity. This is supported by latitudinally-dependent PWC loss risk changes on comfort days and by greater increase in PWC loss risks on dangerous days than on discomfort days. This result suggests that the impacts of global warming on the PWC loss risks are regionally uneven, highlighting the importance of meteorological aspects in designing region-specific adaptation policies to address the PWC loss risks due to heat stress.

While this study provides useful information on the PWC loss risks, there are still many areas for improvement. The PWC formula used in this study is based on group-level data and may not fully account for individual physical differences and working environments (Foster et al. 2021; Dawkins et al. 2023). For instance, PWC losses due to heat stress may vary by gender and age, and may also vary depending on whether the worker is indoors or outdoors (Suorsa et al. 2022; Nelson et al. 2023). In addition, the present study is based on one hour of exposure. Applying the full-day exposure models, such as those proposed by Smallcombe et al. (2022) and Havenith et al. (2024), could enable the integration of cumulative heat exposure effects throughout the day, potentially leading to more accurate assessments of the overall impacts on human productivity and well-being, and informing the development of more effective strategies for mitigating heat stress risks. A more sophisticated index could be also used to quantify heat stress. The Humidex index used in this study estimates heat stress using temperature and humidity, excluding solar radiation and wind speed which are critical for heat stress in outdoor occupations. As a result, the findings of this study may not fully capture the actual heat stress experienced in local outdoor environments. However, the results based on daily maximum WBGT, which incorporates solar radiation and wind speed, show similar spatiotemporal patterns to those based on the Humidex (Figures S1 and S2). The long-term trend in PWC loss risk, primarily driven by the increasing frequency of heat stress days due to global warming, is also comparable. This result supports the present study.

To address these limitations, future research should apply full-day exposure models that incorporate all key climatic variables (e.g., temperature, humidity, solar radiation, and wind) as well as socio-economic and demographic factors (e.g., occupation, work intensity, access to cooling, gender, age, and health). Despite uncertainties in climate model outputs, such efforts are necessary to quantitatively assess heat-related PWC losses under future climate changes in a more comprehensive and realistic manner.

## Supplementary Information

Below is the link to the electronic supplementary material.

Supplementary Material 1

## Data Availability

The data of the fifth generation of the European Centre for Medium-Range Weather Forecast global reanalysis (ERA5) can be accessed at https://cds.climate.copernicus.eu/cdsapp#!/dataset/reanalysis-era5-single-levels?tab=overview. The gridded population dataset in 2010 can be obtained via http://sedac.ciesin.columbia.edu/data/set/popdynamics-1-8th-pop-base-year-projection-ssp-2000-2100-rev01/data-download.
