# Supplementary material for: Increasing physical work capacity losses due to heat stress increase
Source: Int J Biometeorol. 2025 Aug 15;69(11):3073–83. doi: 10.1007/s00484-025-03008-0 (PMC12540639; doi:10.1007/s00484-025-03008-0)
Supplement: Supplementary file 1 — Supplementary Material 1 [file 484_2025_3008_MOESM1_ESM.docx]

**Supporting Information for**

**Increasing physical work capacity losses due to heat stress increase**

Seok-Geun Oh^1*^, Seok-Woo Son^1,2^, and Dong-Chan Hong^1^

^1^School of Earth and Environmental Sciences, Seoul National University, Seoul, South Korea

^2^Climate Technology Center, Seoul National University, Seoul, South Korea

Contents of this file: Figures S1 to S2

***Corresponding author:** Seok-Geun Oh (seokgeunoh@snu.ac.kr)


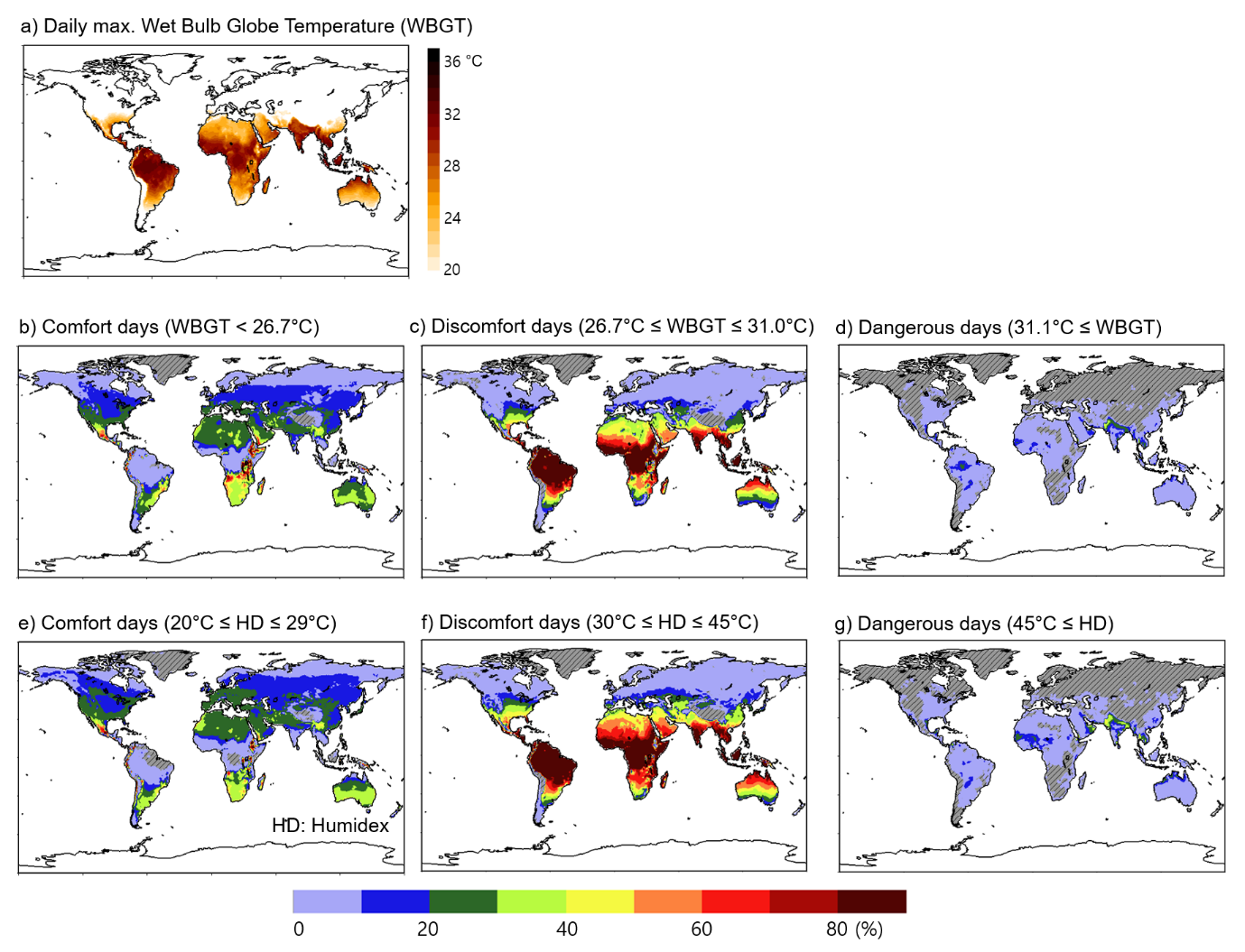


Figure S1. Spatial distribution of (a) daily maximum wet-bulb globe temperature (WBGT) and the probability of (b–d) comfort, discomfort, and dangerous days based on WBGT for the period 1985–2023. For comparison, the probability of (e–g) heat stress days derived from the Humidex (HD) index is also presented.


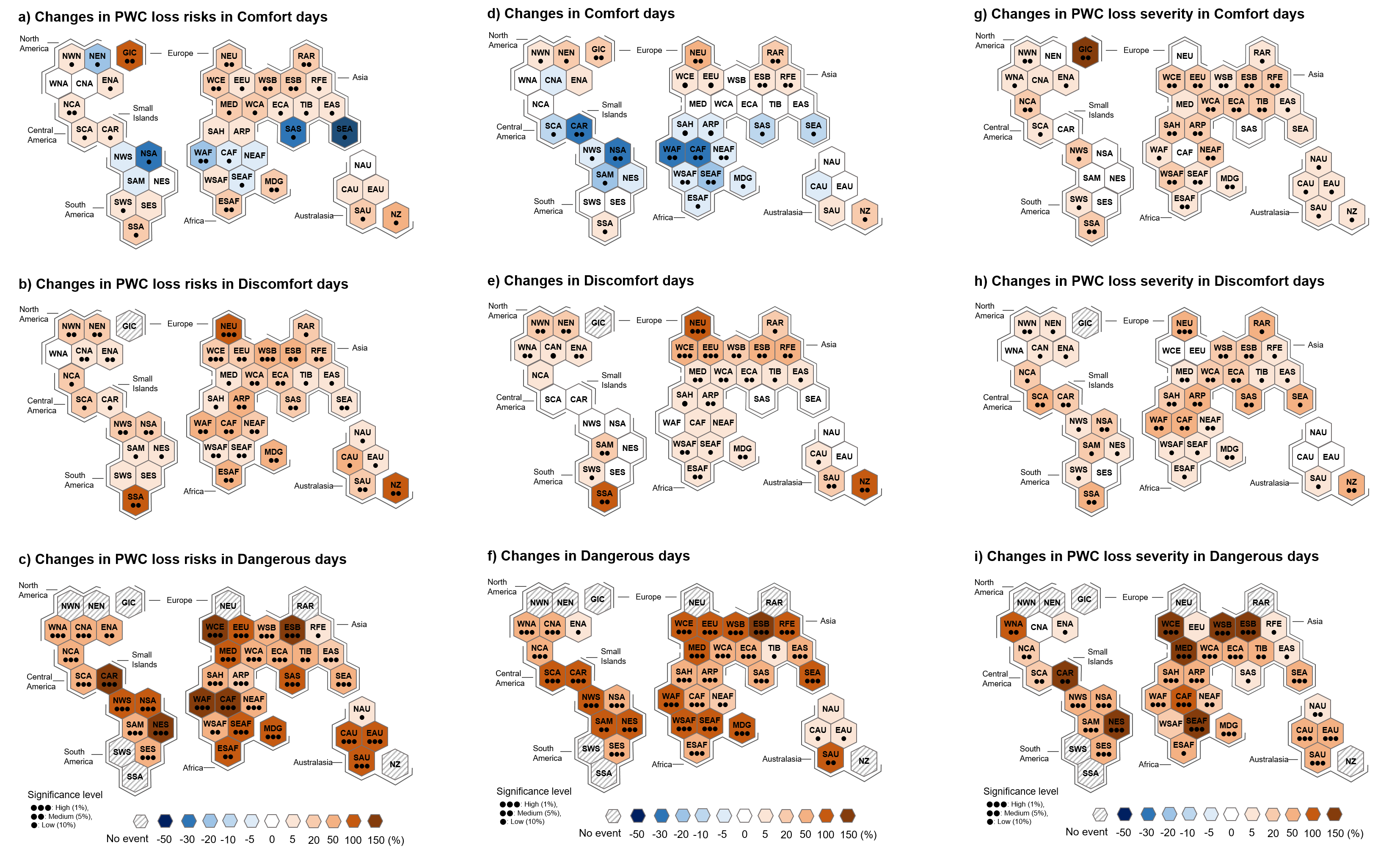


Figure S2. Same as Figures 3, 5, and 6 in the main text, but based on calculations using the web-bulb globe temperature (WBGT) index.
